# Hidden risk patterns among acute ischemic stroke patients identified by latent class analysis

**DOI:** 10.3389/fneur.2025.1597361

**Published:** 2025-07-29

**Authors:** Lei Gu, Zhezhe Sun, Xiangqing Hou, Wanli Zhang, Binbin Deng, Xiaoyang Chen, Xiang Li, Yifan Cheng

**Affiliations:** ^1^The Department of Rehabilitation Medicine, Ningbo Medical Center Lihuili Hospital, Ningbo, China; ^2^Department of Neurology, The First Affiliated Hospital of Ningbo University, Ningbo, China; ^3^Guangzhou National Laboratory, Guangzhou International Bio Island, Guangzhou, China; ^4^Department of Neurology, The First Affiliated Hospital of Wenzhou Medical University, Wenzhou, China; ^5^Department of Rehabilitation Medicine, Yiwu Rehabilitation Hospital, Jinghua, China; ^6^Department of Rehabilitation Medicine, Xiang'an Hospital of Xiamen University, Xiamen, China; ^7^Center for Rehabilitation Medicine, Department of Neurology, Zhejiang Provincial People’s Hospital, Affiliated People’s Hospital, Hangzhou Medical College, Hangzhou, China

**Keywords:** acute ischemic stroke, latent class analysis, subgroups, risk patterns, poor outcomes

## Abstract

**Background:**

Few studies focus on the comprehensive influence of multiple risk factors on the follow-up outcomes of acute ischemic stroke (AIS) patients. To fill this gap, this study aims to identify different subgroups with specific clinical characteristics and risk patterns among patients with AIS and to provide individualized treatment plans accordingly.

**Methods:**

We obtained clinical follow-up data from 448 AIS patients within 72 h of admission. Subgroup patients were characterized by latent class analysis (LCA) using 5 risk factors of AIS. Cox proportional hazard regression analysis was used to explore the relationship between classified risk factor patterns and functional outcomes of patients with AIS at 3 months.

**Findings:**

We obtained two risk factor patterns as “Elderly with low lymphocytes,” and “Participants with low neutrophils and high lymphocytes.” Class 1 (*n* = 214, 47.8%) had lower lymphocytes levels and was mainly elderly. Patients in Class 2 (*N* = 234, 53.2%) had higher lymphocytes levels and lower neutrophils levels than those in Class 1. In addition, CRP levels were mostly low in both Classes 1 and 2. There was a significant difference in poor functional outcomes between the two patterns after adjusting various confounders (*p* < 0.001). Compared with patients in Class 2, patients in Class 1 had a higher risk of adverse functional outcomes (adjusted Hazard Ratio, 3.21; 95% confidence interval: 2.07–4.98; *p* < 0.001).

**Interpretation:**

In our study, LCA was used to identify a 2-Class LCA model that was shown distinct clinical features and laboratory measurements among AIS patients. Our findings are beneficial for health management and therapy.

## Introduction

Stroke is one of the most serious causes of death and disability around the world, and the number of new cases in China reaches 3.94 million every year (1, 2). It is estimated that 5.5 million deaths and 44 million physical disabilities worldwide attribute to stroke yearly; therefore, stroke has currently become one of the most devastating neurological diseases which brings huge health burdens around the world (3). The latest Global Burden of Disease (GBD) analysis (1990–2019) reported a significant rise in the annual global count of strokes and stroke-related deaths, despite major reductions in age-standardized stroke rates. These declines were most pronounced in individuals aged 70 years or older, underscoring a paradox where improved age-specific outcomes coexisted with growing absolute burdens due to population growth and aging (4). In 2021, stroke was the third most common GBD level cause of death (5). Specifically, ischemic stroke accounts for 70–80% of all stroke cases (6). The neurological damage sustained by patients after a stroke is often severe and long-lasting, and the high disability rate attributed to stroke imposes significant physical and mental burdens on caregivers (7). Consequently, developing methods to classify acute ischemic stroke (AIS) patients for prognostic evaluation has become an urgent priority for families and society as a whole.

Numerous studies have demonstrated that advanced age is strongly associated with poor prognosis in patients with acute ischemic stroke (AIS) (8, 9). For instance, one study found that leukocytosis at admission correlates with increased stroke severity and poorer clinical outcomes (10). Furthermore, evidence confirms that elevated neutrophil counts and reduced lymphocyte levels are linked to worse functional outcomes in acute cerebral infarction (11). Additionally, research has highlighted that cholesterol and C-reactive protein (CRP) levels are also predictive of clinical prognosis in AIS patients (12). However, prior investigations have predominantly focused on individual risk factors in isolation, with limited exploration of how the combined influence of multiple biomarkers impacts outcomes in AIS populations.

Latent class analysis (LCA) is a widely used unsupervised, model-based clustering approach for identifying categorical latent variables using categorical observed indicators. This approach clusters individuals into subgroups (latent classes) based on shared patterns of multiple risk factors, minimizing within-group heterogeneity while ensuring distinct separation between classes. These subgroups reveal specific risk profiles associated with disease progression or outcomes. To date, few studies have employed LCA to explore how distinct risk factor combinations influence the prognosis of acute ischemic stroke (AIS) patients. To address this gap, our study aimed to: (1) identify and characterize latent risk patterns among AIS patients based on multiple clinical and biomarker variables; (2) investigate the associations between hidden risk patterns and adverse functional outcomes.

## Methods

### Study population

This study was conducted at a tertiary care hospital in Wenzhou, China. Clinical and laboratory data were retrospectively collected from the hospital’s prospective stroke registry between October 2017 and September 2018. Participants included newly diagnosed adult patients with acute ischemic stroke (AIS) who received treatment within 72 h of symptom onset. Inclusion criteria required confirmation of AIS through clinical symptoms and neuroimaging (CT/MRI) as defined by the World Health Organization (13). The study protocol was approved by the Ethics Committee (Institutional Review Board) of the First Affiliated Hospital of Wenzhou Medical University, and written informed consent was obtained from all participants or their legal guardians.

### Data collection procedures

All eligible patients meeting the inclusion criteria were consecutively enrolled in this prospective cohort study. The following exclusion criteria were applied: (1) patients who received thrombolytic therapy and mechanical endovascular therapy; (2) patients who met clinical criteria for acute and chronic infection of the lung, urinary tract, or other sites or laboratory results indicating such infections; (3) patients who had surgery, severe trauma, or burns within 3 months before admission; (4) patients with malignant tumors, blood diseases, or rheumatoid immune-related diseases; (5) patients undergoing steroid or immunosuppressant treatment; and (6) patients with severe liver or renal impairment.

It would be better to have another external validation dataset to assess the performance of the present model. Therefore, we obtained key variables of AIS patients as external validation from The National Health and Nutrition Examination Survey (NHANES) datasets from four waves of data collection (2015–2016, 2017–2018, 2019–2020, 2021–2023). NHANES has been examining a nationally representative sample of the US population since 1999, continuously collecting health-related data. The diagnosed of AIS was defined as a positive answer to the question “*Has a doctor or other health professional ever told {you/SP} that {you/s/he}.had a stroke?*”

The baseline clinical data were collected within 72 h of admission, including (1) demographic data such as age, sex, length of hospital stay, body mass index, smoking and drinking behavior. (2) Complications such as hypertension, diabetes, hyperlipidemia, and ventricular fibrillation. (3) Drugs medication. In the study, hypertension was defined as having a recurring systolic blood pressure of 140 mmHg or higher and/or a diastolic blood pressure of 90 mmHg or higher, either by a prior diagnosis or using antihypertensive medications. Diabetes was confirmed with fasting blood glucose levels of 7 mmol/L or higher, random blood glucose of 11.1 mmol/L or higher, or glycosylated hemoglobin levels of 6.5% or higher, in addition to a history of being diagnosed with diabetes or using diabetes drugs. Hyperlipidemia was defined as having serum total cholesterol levels of 5.2 mmol/L or higher, triglyceride levels of 1.7 mmol/L or higher, low-density lipoprotein cholesterol levels of 3.4 mmol/L or higher, or high-density lipoprotein cholesterol levels of <1.0 mmol/L, or a prior diagnosis of hyperlipidemia. Atrial fibrillation was diagnosed based on definitive ECG data or any previously known episode of AF. Smoking was defined as smoking more than one cigarette per day for 6 months or longer. Average daily alcohol consumption of 2 U for men and 1 U for women was considered to be alcohol consumption. The use of antiplatelet agents, anticoagulants, or statins was recorded at discharge.

Laboratory data obtained on admission included complete blood counts and other laboratory parameters such as total cholesterol, triglycerides, CRP, low-density lipoprotein, high-density lipoprotein, serum creatinine, blood urea nitrogen, uric acid, homocysteine, fasting blood glucose, postprandial blood glucose, and glycosylated hemoglobin. The results of all tests were provided by the Laboratory Department of the hospital. Additionally, stroke severity on admission was assessed using the NIHSS National Institutes of Health Stroke Scale (14).

The follow-up work was completed by two experienced neurologists in the outpatient department through telephone interviews, therefore, they can doubled ascertain the validity of the data. We assessed functional recovery after 3 months in patients with AIS using the modified Rankin Scale (mRS, with scores ranging from 0 to 6) (15). Follow-up was terminated at the expiration of 3 months after AIS or when the death occurred. An mRS ≥ 3 was defined as a poor functional outcome and a favorable functional outcome was mRS < 3 (16).

### Latent class analysis (17)

Latent class analysis is an approach used to identify novel subgroup AIS patients with similar clinical characteristics based on their responses to a set of categorical indicator variables. The subgroups are referred to “latent class” which can be utilized to detect unobserved heterogeneity in samples. In this study, the number of subgroups with optimal performance for data was determined using the lowest BIC criteria (18, 19). Besides, the multiple comparisons between different classes were also conducted by the bootstrap likelihood ratio test (BLRT) and Lo–Mendell–Rubin likelihood ratio test (LMR). The entropy was additionally utilized to measure separation performance between latent classes. Model simplicity and clinical interpretability were also taken into consideration when we determining the best “latent classes.”

### Statistics analysis

Categorical variables in this study were expressed as frequencies and proportions, and comparisons between categorical variables were completed using Chi-square tests. In this study, the missing percent of all variables were <10% (Supplementary Table 1). Therefore, we employ a commonly used missing data imputation approach, utilizing the median for continuous variables. The result suggested that there is no obvious difference between imputed before and after. For the variable selection, Univariate Cox regression model and linear regression model with Least Angle Regression (LAR) through 10-fold cross-validation were used to perform variable selection; Then, multivariate Cox regression was performed to obtain significant biomarkers for establishing a clustering model. To assesses potential bias in estimating regression parameters in a logit model, the most likely class revealed by LCA was used as the true class. In this study, the model selection was conducted based on the lowest BIC criteria which have been widely used in other similar studies (18, 19). Besides, the clinical interpretation had also been carefully considered since we identified two major clusters with obvious separation and divided all AIS patients into two groups (low, high) risk of poor outcomes. The 2-classes model in this study can be very straightforward in clinical practice, it not only offered additional interpret for distinct clinical features and laboratory measurements among AIS patients but also have good homogeneity in the same classes. Finally, an optimal 2-class latent class model was selected based on characteristics (Age) and laboratory data (neu). This model had the lowest Bayesian information criterion (BIC), Akaike information criterion (AIC), the “consistent AIC” (CAIC), and the adjusted BIC using *Rissanen’s* sample size adjustment (SSA-BIC) and was clinically interpretable, which supports the best performance and stability of the model. Variable time-to-event comparisons were performed using the log-rank test. In addition, Cox proportional hazards regression models were used to assess the relationship between the outcome and the risk factor pattern based on LCA subgroups. Moreover, a multivariate model was established to adjust for some potential confounders, with *p* < 0.05 in univariate analysis, including atrial fibrillation (AF), hemoglobin (HB), albumin (ALB), total protein (TLB), triiodothyronine (T3), thyroxine (T4) and prothrombin time (PT). Hazard ratios and 95% confidence intervals (HR, 95% CI) were used to illustrate the relative risk.

This study used the National Health and Nutrition Examination Survey (NHANES) datasets from four waves of data collection (2015–2016, 2017–2018, 2019–2020, 2021–2023) as external data. To enhance comparability between the training data and external data, we performed propensity score matching (PSM). Propensity scores were estimated via logistic regression using baseline covariates common to both datasets (age, Lymphocytes, Neutrophils, CRP, and Cholesterol). We matched training and external subjects 1:1 without replacement using a caliper of 0.1 SD of the logit propensity score. Standardized mean differences for all covariates were <0.05 after matching except CRP, indicating adequate balance. Unmatched external subjects were excluded from analysis.

A two-sided *p* value of 0.05 was considered statistically significant. The figures were plotted using R-Studio 1.2.5001 (Copyright 2009–2018 R-Studio, Inc.). All data management and statistical analysis were performed using SAS 9.4 (Copyright, 2002–2012, SAS Institute Inc., NC). The LCA model was performed using *PROC LCA* in SAS 9.4.

## Results

### Baseline characteristics of the study subject

A total of 448 subjects were included and entered the final analysis. The average age was 66.8 (± 12.2) years old, and 58.48% of participants were more than 65 years old. The ratio of male to female was 65%/35%. The proportion of drinker and smoker among all patients were 31.7 and 42.9%, separately. Nearly 82% of participants were diagnosed as hypertension, while 38.4% of subjects suffered from diabetes and 38.9% suffered from dyslipidemia in this study. Moreover, details about baseline characteristics of patients were shown in the Supplementary Table 2.

### Feature selection by cox regression model and least-angle regression

For the variable selection, firstly of all, Univariate Cox regression model was used to select 17 significant risk factors of AIS (Supplementary Table 3); Furthermore, linear regression model was used to perform variable selection with Least Angle Regression (LAR) and 10-fold cross-validation, and an optimal model including 7 risk factors was obtained as their good performance through cross-validation (Supplementary Figure 1); Finally, we performed multivariate Cox regression based on the 7 risk factors and to obtain five significant biomarkers for establishing a clustering model (Supplementary Table 3).

### Characteristics of risk factor patterns

According to their risk factor characteristics and the model fit statistics (Table 1), the 2-Class model has a good clinical interpretation and significantly better fit statistics than 1 or 3 or 4 classes models; Moreover, the results (Supplementary Table 4) suggest that the BLRT and LMR differed for the model consisting of 2 and 3 classes, so preference was given to the significant BLRT and LMR of the 2 or 3-class model. Moreover, based on the criteria of Occam’s Law of Razor (20), the superiority model for achieving optimal results is a model with fewer variables, thus, the 2-class model can be more straightforward in clinical practice. Moreover, the results shown distinct 2 clusters in multivariate space, so preference was given to the 2-class model which is consistent with the proposed model (Supplementary Figure 2). Therefore, the results also support that the LCA model with 2 classes is acceptable and our findings are reliable. Class 1 is most likely for older adults with low cholesterol. Class 2 is characteristics by high lymphocytes and low neutrophils (Table 2). Compared with Class 1, the LC level of the patients in Class 2 was higher, the NC level was lower, and the cholesterol level was significantly higher.

**Table 1:** Model fit statistics of latent class analysis.

K-class	AIC	BIC	CAIC	SSA-BIC	Entropy
1	70.37	90.89	95.89	75.02	1.00
2	44.87	90.03	101.03	55.12	0.37
3	49.89	119.67	136.67	65.72	0.56
4	54.32	148.73	171.73	75.74	0.63

AICm, Akaike information criterion; BIC, Bayesian Information Criterions; The “consistent AIC,” CAIC; The adjusted BIC using Rissanen’s sample size adjustment, SSA-BIC.

**Table 2:** Characteristic of different patterns.

Variables	Total *N* = 448	Class 1 *N* = 214	Class 2 *N* = 234	*P*-value
LCA variables				
Age, years				<0.001
≤65	186 (41.52)	54 (25.2)	132 (56.4)	
>65	262 (58.48)	160 (74.8)	102 (43.6)	
Neutrophils, 10^9^ cells/L				<0.001
≤5.6	328 (73.21)	132 (61.7)	196 (83.8)	
>5.6	120 (26.79)	82 (38.3)	38 (16.2)	
Lymphocytes, 10^9^ cells/L				<0.001
≤1.7	243 (54.24)	213 (99.5)	30 (12.8)	
>1.7	205 (45.76)	1 (0.5)	204 (87.2)	
Chol, mmol/L				<0.001
≤4.3	198 (44.20)	125 (58.4)	73 (31.2)	
>4.3	250 (55.80)	89 (41.6)	161 (68.8)	
CRP, mg/L				0.013
≤6.7	391 (87.28)	178 (83.2)	213 (91.0)	
>6.7	57 (12.72)	36 (16.8)	21 (9.0)	
Non-LCA variables				
Sex				0.624
Male	290 (64.73)	141 (65.9)	149 (63.7)	
Female	158 (35.27)	73 (34.1)	85 (36.3)	
Intensive care				0.938
No	445 (99.3)	212 (99.1)	233 (99.6)	
Yes	3 (0.7)	2 (0.9)	1 (0.4)	
Lesions location				0.335
Frontal lobe	20 (4.5)	11 (5.1)	9 (3.8)	
Parietal lobe	29 (6.5)	14 (6.5)	15 (6.4)	
Temporal lobe	13 (2.9)	9 (4.2)	4 (1.7)	
Occipital lobe	34 (7.6)	14 (6.5)	20 (8.5)	
Insula	22 (4.9)	10 (4.7)	12 (5.1)	
Pons/ Medulla oblongata	66 (14.7)	25 (11.7)	41 (17.5)	
Cerebellum	32 (7.1)	12 (5.6)	20 (8.5)	
Basal ganglia	98 (21.9)	53 (24.8)	45 (19.2)	
Others	134 (29.9)	66 (30.8)	68 (29.1)	
Main clinical manifestation				0.134
Dizziness	18 (4.0)	9 (4.2)	9 (3.8)	
Hemiplegia/Limb weakness	266 (59.4)	124 (57.9)	142 (60.7)	
Slurred speech	31 (6.9)	19 (8.9)	12 (5.1)	
LCA variables				
Numbness	89 (19.9)	48 (22.4)	41 (17.5)	
Difficulty walking	27 (6.0)	8 (3.7)	19 (8.1)	
Others	17 (3.8)	6 (2.8)	11 (4.7)	
Atrial fibrillation				0.005
No	395 (88.17)	179 (83.6)	216 (92.3)	
Yes	53 (11.83)	35 (16.4)	18 (7.7)	
Anticoagulant				0.051
No	408 (91.07)	189 (88.3)	219 (93.6)	
Yes	40 (8.93)	25 (11.7)	15 (6.4)	
Smoking				0.275
No	256 (57.1)	128 (59.8)	128 (54.7)	
Yes	192 (42.9)	86 (40.2)	106 (45.3)	
Drinking				0.111
No	306 (68.3)	154 (72.0)	152 (65.0)	
Yes	142 (31.7)	60 (28.0)	82 (35.0)	
Comorbidities				
Hypertension	367 (81.9)	178 (83.2)	189 (80.8)	0.508
Diabetes	172 (38.4)	74 (34.6)	98 (41.9)	0.112
Dyslipidemia	174 (38.9)	63 (29.1)	111 (47.6)	<0.001
Monocyte	0.5 (0.4, 0.7)	0.5 (0.4, 0.7)	0.5 (0.4, 0.7)	0.862
HB	137.0 (126.0, 146.0)	133.0 (121.0, 144.0)	139.0 (130.0, 148.0)	<0.001
WBC	6.9 (5.7, 8.2)	6.8 (5.5, 8.3)	6.9 (5.8, 8.2)	0.221
TLB	66.7 (63.6, 69.9)	66.0 (62.7, 69.3)	67.4 (64.5, 70.4)	0.003
ALB	37.8 (35.4, 39.6)	36.8 (34.4, 38.7)	38.5 (36.7, 40.2)	<0.001
T3	4.4 (4.0, 4.8)	4.3 (3.8, 4.6)	4.6 (4.2, 4.9)	<0.001
T4	11.1 (10.1, 12.3)	11.3 (10.3, 12.5)	11.0 (9.8, 12.1)	0.008
PT	13.5 (13.0, 14.0)	13.7 (13.1, 14.2)	13.4 (12.9, 13.9)	0.003
Tbil	11.0 (8.0, 14.0)	11.0 (8.0, 14.0)	11.0 (9.0, 14.0)	0.513
Direct bilirubin	4.0 (3.0, 5.0)	4.3 (3.0, 6.0)	4.0 (3.0, 5.0)	0.070

HB, Hemoglobin; WBC, white blood cells; TLB, total protein; ALB, albumin; Tbil, bilirubin.

Finally, 48% of participants were assigned to Class 1, while the remaining to Class 2 (52%). As shown in Table 3, categorical features are like the probability distributions computed by LCA. Most patients in Class 1 were older people, since 74.8% of them >65 years. Moreover, most patients in Class 2 were low neutrophils (83.8%) and high lymphocytes levels (87.2%). In comparison with patients in Class1 Cholesterol levels were higher in Class 2 (68.8%). In addition, the proportion of CRP levels and neutrophils were similar in Class 1 and 2, although their difference between two classes achieved statistically significant.

**Table 3:** Conditional probabilities of patients with risk factor patterns and outcomes.

Variable	Class 1	Class 2
Age, years		
≤65	0.2662	0.5252
>65	0.7338	0.4748
Neutrophils, 10^9^ cells/L		
≤5.6	0.5980	0.8312
>5.6	0.4020	0.1688
Lymphocytes, 10^9^ cells/L		
≤1.7	0.8660	0.3034
>1.7	0.1340	0.6966
Chol, mmol/L		
≤4.3	0.5922	0.3310
>4.3	0.4078	0.6690
CRP, mg/L		
≤6.7	0.8113	0.9182
>6.7	0.1887	0.0818
Latent class probabilities	0.4246	0.5754

### Association of risk factor pattern with follow-up outcomes

We further ruled out confounding effects in this risk factor model. There was significance (*p* < 0.001) associations between the poor prognosis outcomes of patients and risk patterns. Specifically, 42.32% of patients who had unfavorable outcomes in Class 1, whereas 11.97% of subjects in Class 2 shown adverse outcomes. Additionally, the HR in the high-risk group was 3.21 (95% CI 2.07–4.98) compared to the low-risk group after adjusting for AF, HB, TLB, Alb, T3, T4, PT, anticoagulant, and direct bilirubin (Table 4), that means the high-risk group exhibiting a 3.21-fold increased hazard of mortality compared to the low-risk group. In the correlation analysis, variable pairs with correlation coefficients ≥0.60 were considered to be closely related, and the potential interactions of the variable pairs should be considered in the final models. The Supplementary Table 5 suggested that the variable pairs of atrial fibrillation and Anticoagulant and ALB and TLB should additionally investigate interactions in the final model. The Table 4 reported that after adjusted all confounding factors and potential interactions, the high-risk group exhibiting a 3.07-fold increased hazard of mortality compared to the low-risk group.

**Table 4:** Association with risk factor patterns and outcomes.

Patterns	*N*	Unfavorable outcomes	Cruded model		Adjusted model 1		Adjusted model 2	
			HR (95% CI)	*P*	HR (95% CI)	*P*	HR (95% CI)	*P*
Class2	234	28	Ref.		Ref.		Ref.	
Class1	214	102	3.86 (2.54, 5.87)	<0.001	3.21 (2.07, 4.98)	<0.001	3.07 (1.98, 4.78)	<0.001

Adjusted model 1 consists of Atrial fibrillation, HB, TLB, Alb, T3, T4, PT, Anticoagulant, Direct bilirubin; Adjusted model 2 consists of Atrial fibrillation, HB, TLB, Alb, T3, T4, PT, Anticoagulant, Direct bilirubin, Atrial fibrillation* Anticoagulant, TLB*Alb.

**Figure 1:**
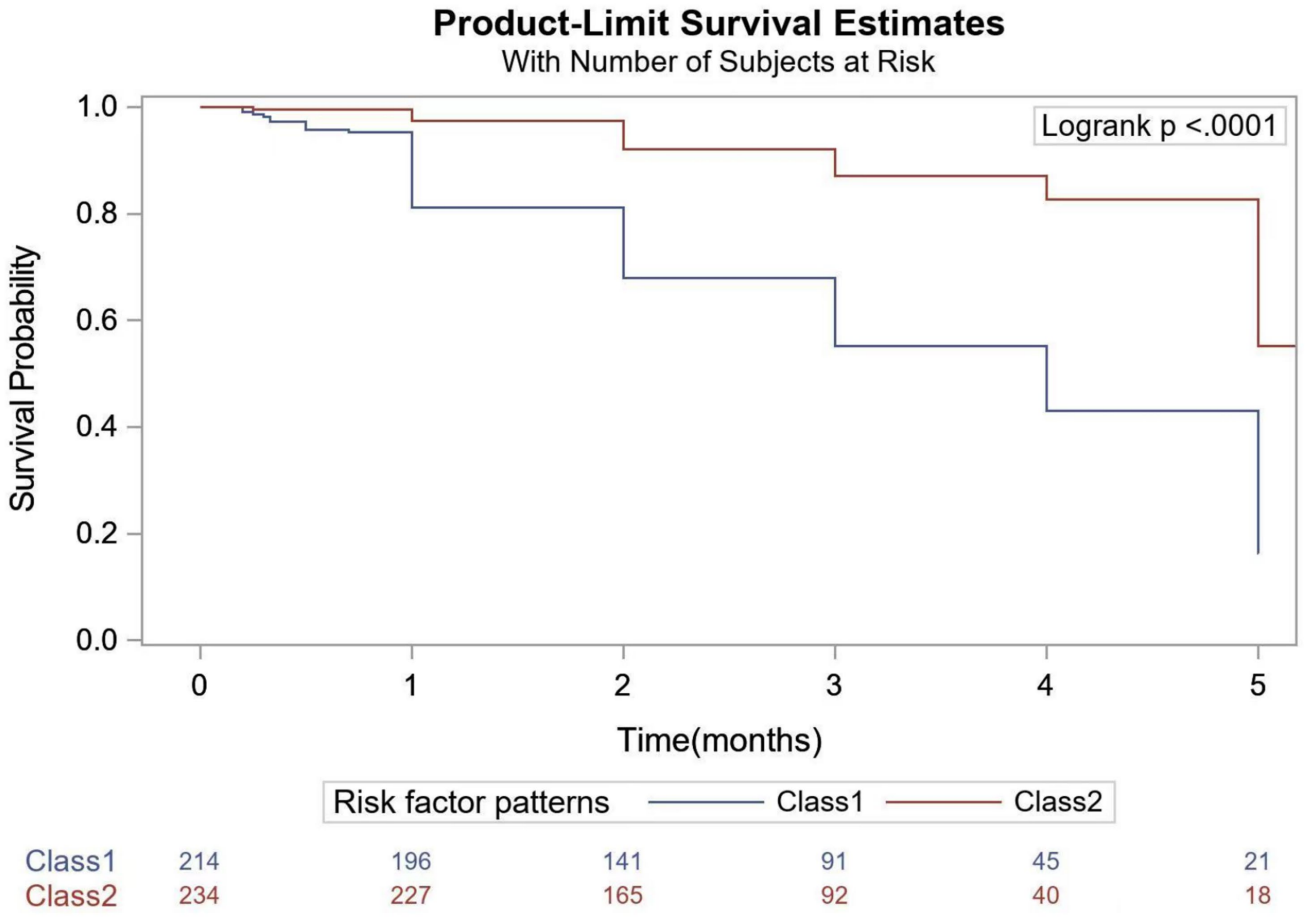
Difference in survival probability.

The Kaplan–Meier curve also suggested that there is a significant difference between high- and low-risk groups. As shown in Figure 1, there was a significant difference in survival times between Class 1 and Class 2 (log rank test *p* < 0.001).

### The external validation of the proposed model

In this study, a total of 1,331 AIS patients from NHANES were included and taken as external validation datasets. To improve the comparable among the training and validation datasets, we used propensity score matching (PSM) to get more balanced comparison between them. Supplementary Table 6 shown that matched datasets had better comparable overall. To re-project the external data into the original training-set UMAP space and visualize whether those points fall within the class clusters identified in the training data, we additionally conducted a UMAP figure based on the external datasets (*n* = 417). Supplementary Figure 3 shown that there has similar trend with Supplementary Figure 2 to divided all participants into 2 clusters.

## Discussion

In this study, we identified two risk factor patterns in AIS patients using LCA. The two patterns were defined as the “elderly group with low lymphocyte” and the “high lymphocyte, low neutrophil group.” Patients in the Class1 pattern had a significantly higher risk of adverse outcomes than those in the Class 2 pattern (*p* < 0.05). Our study also revealed that compared with Class1, the current hypercholesterolemia group was dominated by the “high lymphocytes, low neutrophils group” and had a better prognosis, which suggesting a paradox for people with high cholesterol.

LCA was employed to identify distinct risk factor profiles in AIS patients. This method not only accounts for the interdependencies among multiple risk factors but also enables the exploration of disease-specific risk patterns. A key feature of LCA is its treatment of external variables as independent across different levels of the latent class hierarchy (21). By leveraging the combined characteristics of multiple risk factors (e.g., demographic, biochemical, and inflammatory markers), LCA classifies patients into latent subgroups with shared risk profiles. For example, a prior study using latent class growth analysis (LCGA) categorized 367 first-time AIS patients in Taiwan into five functional recovery trajectories, each associated with distinct prognostic outcomes (22). Similarly, another study applied LCA to stratify 399 Australian stroke survivors into two health risk groups based on modifiable risk factors, revealing significant differences in physical function and independent living capacity (23). However, to date, few studies have utilized LCA to investigate risk factor clustering in Chinese AIS populations, highlighting the novelty of our approach.

In our study, we defined age, neutrophil, lymphocyte, cholesterol, and CRP as risk factors for poor prognosis in AIS. To explore the internal relationship among multiple factors, this study used LCA to divide AIS patients into different groups based on specific risk factors pattern. Finally, we obtained two-class model which shows good statistical fits and clinical interpretation than the LCA model with 1, 3 and 4 clusters. For Class 1, patients had a significantly higher age and low LYM who had worse prognosis than patients in Class 2. According to previous studies, aging may increase the risk of adverse outcomes and CRP levels. Furthermore, many studies have demonstrated that CRP levels can significantly increase the risk of death and MACCE (12). Similar results were also found in our study. It is suggested that elderly patients with AIS and high CRP should pay more attention to daily health care.

The high lymphocyte-to-neutrophil ratio (LNR) reflects a maladaptive immune milieu, combining excessive adaptive immune-mediated neuroinflammation with compromised innate defense, increasing secondary injury and infection risks, ultimately leading to poorer functional outcomes. Neuroinflammation is now recognized as a pivotal contributor to the development and progression of ischemic stroke. Peripheral neutrophils exacerbate injury through mechanisms such as the release of matrix metalloproteinase-9 (MMP-9), which disrupts the blood–brain barrier and worsens outcomes, and the production of free oxygen radicals that directly damage brain tissue. In contrast, lymphocytes often exhibit neuroprotective properties, promoting neurological recovery by modulating inflammatory responses and supporting tissue repair. The neutrophil-to-lymphocyte ratio (NLR) emerges as a critical biomarker, integrating these opposing immune pathways to offer a more nuanced understanding of neuroinflammatory dynamics in ischemic stroke. By reflecting the balance between destructive neutrophil-driven processes and protective lymphocyte activity, NLR enhances prognostic insights and underscores the dual role of immune mechanisms in stroke pathology (24–26).

The characteristics of the risk factors in the two risk factor models show that the current hypercholesterolemia group is dominated by the “high lymphocytes, low neutrophils group,” accounting for 79.3%. This pattern has a better prognosis than the other, suggesting a paradox for people with high cholesterol. Several studies have suggested similar results. There are several reasons for this paradox. First, cholesterol is an important element in cell membranes, intracellular transport, and cell signaling, and low cholesterol levels can alter membrane fluidity, resulting in the failure of neuronal cells to resist local hyperosmolarity and acidosis under ischemic stress (27–29). Second, low cholesterol levels reduce the synthesis of stress hormones, which reduces the body’s ability to cope with an acute injury. Finally, it was attributed to uncontrollable confounding factors (26–28 years), such as age (30).

Strengths of this study include prospective design (quantitative relationship between indicators and outcomes), and use of life-cycle analysis. Our study also has several limitations. First, our study focused on a single-center risk factor pattern in the Han population, so the lack of large sample size and applicability of these findings to other minority populations is problematic. Second, AIS patients who were excluded from this study and received thrombolytic therapy and mechanical endovascular therapy should also participate in the treatment in the future. Third, some specific lipoproteins such as small-density LDL, electronegative LDL, and lipoprotein(a) were not measured, and we were unable to validate operational mechanisms to explain the identified risk associations. In addition, the effect of statins on previous use of statins was not strictly controlled, which may be one of the reasons for the high cholesterol paradox. Finally, our study assessed short-term MRS only at 3 months. However, previous studies in Taiwan have shown that there was a significant difference in motor function recovery outcomes if MRS is continuously assessed over a one-year period (22). Besides, In this study, the association between higher cholesterol and better outcomes among AIS population likely arises from a combination of confounding factors (e.g., statin use, nutritional status), reverse causality (acute-phase response), and potential neuroprotective roles of cholesterol (31). Rigorous prospective studies measuring pre-stroke cholesterol, lipid subtypes, and statin use are needed to clarify these mechanisms.

In conclusion, our study provides insight into the risk factor patterns of AIS patients and highlights the importance of considering multiple risk factors when evaluating prognosis.

## Data Availability

The raw data supporting the conclusions of this article will be made available by the authors, without undue reservation.
