# Supplementary material for: Hidden risk patterns among acute ischemic stroke patients identified by latent class analysis
Source: Front Neurol. 2025 Jul 29;16:1597361. doi: 10.3389/fneur.2025.1597361 (PMC12339565; doi:10.3389/fneur.2025.1597361)
Supplement: Supplementary file 2 [file Table_2.docx]

%inc 'D:\Program Files\SAS program\maogy93.mac';

proc import datafile="D:\Desk\work\work45_gu\work.xlsx" dbms=xlsx out=work;quit;

data work_1;set work(rename=(var1=group1 var2=group var4=surv_time var5=chuxiezh

var6=fufa var7=death var8=surv_time2 var9=id var10=age var11=sex var12=sbp var13=dbp

var14=hbp var16=xuezhi var17=xinfangcd var18=smoke var19=drink var20=naogeng var21=TOAST

var22=in_nihss var23=out_nihss var24=danhe var37=Talb var38=alb var41=jianxinlsm var42=GT

var43=danhongsu var44=jianjdanhs var45=zhijdanhs var46=n var47=cr var48=crlv var49=n_cr var50=niaosuan

var51=chol var52=tg var62=fpg var63=fpg_2 var66=tongxingb var72=jiazhuangx var79=zuoshinj

var80=zuoshimqnj var81=zuofangnj var82=zuofangzd var83=zuoshijghd var84=zuoshihbhd

var85=zuoshizd var87=zuoshiszqgongn var88=jingdongmyh var89=zuojingnmhd var90=zuojingnmpz

var91=youjingnmhd var92=pingzheng var96=huilvfy var97=platet var98=jaingzhiy var99=kangning var100=RMS_3 var101=BI_RS

var102=sfufa));

run;

data work_2;set work_1;

out_nihss1=out_nihss+0;

danhongsu1=danhongsu+0;

zhijdanhs1=zhijdanhs+0;

jiazhuangx1=jiazhuangx+0;

TSH1=TSH+0;

INR1=INR+0;

zuoshinj1=zuoshinj+0;

zuoshiszqgongn1=zuoshiszqgongn+0;

jingdongmyh1=jingdongmyh+0;

zuojingnmhd1=zuojingnmhd+0;

zuojingnmpz1=zuojingnmpz+0;

youjingnmhd1=youjingnmhd+0;

pingzheng1=pingzheng+0;

height1=var93+0;

weight1=var94+0;

drop ck ck_mb p jianxinlsm gt ca mg IgG IgA IgM C4 group1 id naogeng nlr_ plr_ nlr_plr crlv n_cr huilvfy rms_3 bi_rs sfufa

out_nihss danhongsu zhijdanhs ca c3 jiazhuangx tsh inr zuoshinj zuoshiszqgongn

jingdongmyh zuojingnmhd zuojingnmpz youjingnmhd var93 var94 pingzheng;

rename var3=mrs_score out_nihss1=out_nihss danhongsu1=danhongsu zhijdanhs1=zhijdanhs

jiazhuangx1=jiazhuangx tsh1=tsh inr1=inr zuoshinj1=zuoshinj

zuoshiszqgongn1=zuoshiszqgongn

jingdongmyh1=jingdongmyh zuojingnmhdq=zuojingnmhd

zuojingnmpz1=zuojingnmpz youjingnmhd1=youjingnmhd

weight1=weight height1=height pingzheng1=pingzheng;

run;

/*proc means data=work_2 mean;var xuezhi danhe rbc hb hpv talb alb alt ast jianjdanhs*/

/*n cr niaosuan chol tg ldl hdl na k fpg fpg_2 hb1c tongxingb ft3 ft4 pt aptt zuoshimqnj*/

/*zuofangnj zuofangzd zuoshijghd zuoshihbhd zuoshizd EF out_nihss danhongsu zhijdanhs*/

/*jiazhuangx tsh inr zuoshinj zuoshiszqgongn jingdongmyh zuojingnmhd1 zuojingnmpz*/

/*youjingnmhd pingzheng height weight;run; */

data work_3;set work_2;

if missing(danhe) then danhe=0.5766517;

if missing(RBC) then RBC=0.5766517;

if missing(HB) then HB=135.2823146;

if missing(HPV) then HPV=1.0553824;

if missing(Talb) then Talb=66.1828475;

if missing(alb) then alb=37.5284753;

if missing(alt) then alt=23.2460850;

if missing(ast) then ast=26.1098655;

if missing(jianjdanhs) then jianjdanhs=7.4168618;

if missing(n) then n=6.5622472;

if missing(cr) then cr=77.0815730;

if missing(niaosuan) then niaosuan=300.9572072;

if missing(chol) then chol=4.5140950;

if missing(tg) then tg=2.0445701;

if missing(LDL) then LDL=2.5675566;

if missing(HDL) then HDL=1.0527828;

if missing(Na) then Na=140.0769231;

if missing(K) then K=3.8178054;

if missing(fpg) then fpg=6.0576310;

if missing(fpg_2) then fpg_2=9.4107859;

if missing(Hb1C) then Hb1C=6.5592683;

if missing(tongxingb) then tongxingb=12.6841346;

if missing(fT3) then fT3=4.4135198;

if missing(fT4) then fT4=11.34925;

if missing(PT) then PT=13.7020882;

if missing(APTT) then APTT=37.5616241;

if missing(zuoshimqnj) then zuoshimqnj=31.3765586;

if missing(zuofangnj) then zuofangnj=41.3241895;

if missing(zuoshijghd) then zuoshijghd=10.7431421;

if missing(zuoshihbhd) then zuoshihbhd=10.6084788;

if missing(EF) then EF=64.1601990;

if missing(out_nihss) then out_nihss=3.4112360;

if missing(danhongsu) then danhongsu=11.8341014;

if missing(zhijdanhs) then zhijdanhs=4.5539906;

if missing(jiazhuangx) then jiazhuangx=104.8466355;

if missing(tsh) then tsh=2.5831542;

if missing(inr) then inr=1.1017209;

if missing(zuoshinj) then zuoshinj=48.3391521;

if missing(zuojingnmhd1) then zuojingnmhd1=1.2019417;

if missing(youjingnmhd) then youjingnmhd=1.1282828;

if missing(height) then height=159.8217391;

if missing(weight) then weight=65.8739130;

run;

proc freq data=work_3;tables sex;run;

%macro num(train_pct,validate_pct);

proc sql noprint;

select count(*) into :t_nobs from work_3;

quit;

%let train_obs=%sysevalf(&train_pct.*&t_nobs.);

%let validate_obs=%sysevalf(&validate_pct.*&t_nobs.);

%put &train_obs.;

%put &validate_obs.;

%mend;

%num(.7,.3);

%macro partition(train_obs,validate_obs);

proc surveyselect data=work_3 out=split seed=123

group=(&train_obs.,&validate_obs.);

run;

data train validate;set split;

if groupid=1 then do;drop groupid;output train;end;

if groupid=2 then do;drop groupid;output validate;end;

run;

%mend;

%partition(314,134);

/*Table1*/;

data train1;set train;grp=1;

data validate1;set validate;grp=2;

data total;set train1 validate1;run;

proc contents data=total varnum;run;

/*Table2*/

%macro univariate(train=,variable=,ref=,time=,result=,n=);

proc phreg data=&train;

class &variable.(ref=%sysfunc(quote(&ref.))) ;

model &time.*&result.(0) =&variable./risklimits;

ods output parameterestimates=output&n.;

run;

data tmp&n.(keep=parameter aa P);

set output&n.(keep=parameter HazardRatio HRLowerCL HRUpperCL ProbChiSq);

aa=compress(put(HazardRatio,8.3)||"("||put(hrlowercl,8.3)||','||put(hruppercl,8.3)||")");

p=compress(put(probchisq,8.3));

retain parameter aa P;

run;

proc datasets lib=work noprint;delete output&n.;quit;

%mend;

%macro univariate1(train=,variable=,time=,result=,n=);

proc phreg data=&train;

model &time.*&result.(0) =&variable./risklimits;

ods output parameterestimates=output&n.;

run;

data tmp&n.(keep=parameter aa P);

set output&n.(keep=parameter HazardRatio HRLowerCL HRUpperCL ProbChiSq);

aa=compress(put(HazardRatio,8.3)||"("||put(hrlowercl,8.3)||','||put(hruppercl,8.3)||")");

p=compress(put(probchisq,8.3));

retain parameter aa P;

run;

proc datasets lib=work noprint;delete output&n.;quit;

%mend;

%univariate(train=work_3,variable=toast,ref=1,time=surv_time,result=group,n=1);

%univariate1(train=work_3,variable=age,time=surv_time,result=group,n=1);

%univariate1(train=work_3,variable=sex,time=surv_time,result=group,n=2);

%univariate1(train=work_3,variable=sbp,time=surv_time,result=group,n=3);

%univariate1(train=work_3,variable=dbp,time=surv_time,result=group,n=4);

%univariate1(train=work_3,variable=hbp,time=surv_time,result=group,n=5);

%univariate1(train=work_3,variable=dm,time=surv_time,result=group,n=6);

%univariate1(train=work_3,variable=xuezhi,time=surv_time,result=group,n=7);

%univariate1(train=work_3,variable=xinfangcd,time=surv_time,result=group,n=8);

%univariate1(train=work_3,variable=smoke,time=surv_time,result=group,n=9);

%univariate1(train=work_3,variable=drink,time=surv_time,result=group,n=10);

%univariate1(train=work_3,variable=danhe,time=surv_time,result=group,n=11);

%univariate1(train=work_3,variable=rbc,time=surv_time,result=group,n=12);

%univariate1(train=work_3,variable=hb,time=surv_time,result=group,n=13);

%univariate1(train=work_3,variable=wbc,time=surv_time,result=group,n=14);

%univariate1(train=work_3,variable=nc,time=surv_time,result=group,n=15);

%univariate1(train=work_3,variable=lc,time=surv_time,result=group,n=16);

%univariate1(train=work_3,variable=plt,time=surv_time,result=group,n=17);

%univariate1(train=work_3,variable=hpv,time=surv_time,result=group,n=18);

%univariate1(train=work_3,variable=talb,time=surv_time,result=group,n=19);

%univariate1(train=work_3,variable=alb,time=surv_time,result=group,n=20);

%univariate1(train=work_3,variable=alt,time=surv_time,result=group,n=21);

%univariate1(train=work_3,variable=ast,time=surv_time,result=group,n=22);

%univariate1(train=work_3,variable=jianjdanhs,time=surv_time,result=group,n=23);

%univariate1(train=work_3,variable=n,time=surv_time,result=group,n=24);

%univariate1(train=work_3,variable=cr,time=surv_time,result=group,n=25);

%univariate1(train=work_3,variable=niaosuan,time=surv_time,result=group,n=26);

%univariate1(train=work_3,variable=chol,time=surv_time,result=group,n=27);

%univariate1(train=work_3,variable=tg,time=surv_time,result=group,n=28);

%univariate1(train=work_3,variable=ldl,time=surv_time,result=group,n=29);

%univariate1(train=work_3,variable=hdl,time=surv_time,result=group,n=30);

%univariate1(train=work_3,variable=na,time=surv_time,result=group,n=31);

%univariate1(train=work_3,variable=k,time=surv_time,result=group,n=32);

%univariate1(train=work_3,variable=fpg,time=surv_time,result=group,n=33);

%univariate1(train=work_3,variable=fpg_2,time=surv_time,result=group,n=34);

%univariate1(train=work_3,variable=hb1c,time=surv_time,result=group,n=35);

%univariate1(train=work_3,variable=crp,time=surv_time,result=group,n=36);

%univariate1(train=work_3,variable=tongxingb,time=surv_time,result=group,n=37);

%univariate1(train=work_3,variable=ft3,time=surv_time,result=group,n=38);

%univariate1(train=work_3,variable=ft4,time=surv_time,result=group,n=39);

%univariate1(train=work_3,variable=pt,time=surv_time,result=group,n=40);

%univariate1(train=work_3,variable=aptt,time=surv_time,result=group,n=41);

/*%univariate1(train=Train,variable=zuoshimqnj,time=surv_time,result=group,n=42);*/

/*%univariate1(train=Train,variable=zuofangnj,time=surv_time,result=group,n=43);*/

/*%univariate1(train=Train,variable=zuofangzd,time=surv_time,result=group,n=44);*/

/*%univariate1(train=Train,variable=zuoshijghd,time=surv_time,result=group,n=45;*/

/*%univariate1(train=Train,variable=zuoshihbhd,time=surv_time,result=group,n=46);*/

/*%univariate1(train=Train,variable=zuoshizd,time=surv_time,result=group,n=47);*/

/*%univariate1(train=Train,variable=ef,time=surv_time,result=group,n=48);*/

%univariate1(train=work_3,variable=platet,time=surv_time,result=group,n=42);

%univariate1(train=work_3,variable=jaingzhiy,time=surv_time,result=group,n=43);

%univariate1(train=work_3,variable=kangning,time=surv_time,result=group,n=44);

%univariate1(train=work_3,variable=danhongsu,time=surv_time,result=group,n=45);

%univariate1(train=work_3,variable=zhijdanhs,time=surv_time,result=group,n=46);

%univariate1(train=work_3,variable=tsh,time=surv_time,result=group,n=47);

%univariate1(train=work_3,variable=zuoshinj,time=surv_time,result=group,n=48);

%univariate1(train=work_3,variable=pingzheng,time=surv_time,result=group,n=49);

%univariate1(train=work_3,variable=tsh,time=surv_time,result=group,n=50);

%univariate1(train=work_3,variable=toast,time=surv_time,result=group,n=51);

data table2_univar;

set tmp1-tmp51;

run;

proc datasets lib=work noprint;delete tmp1-tmp51;quit;

data table2_univar1;set table2_univar;p0=p+0;drop p;if p0=0 then p0=0.001;run;

data table2_nosig;set table2_univar1;if p0 gt 0.05;run;

data table2_sig;set table2_univar1;if p0 le 0.05;run;

/*age xinfangcd danhe hb wbc nc lc talb alb chol crp ft3 ft4 pt kangning danhongsu zhijdanhs*/

proc glmselect plots=all data=work_3 seed=123;

model group=age xinfangcd danhe hb wbc nc lc talb alb chol crp ft3 ft4

pt kangning danhongsu zhijdanhs

/selection=LAR(choose=cv stop=none) cvmethod=random(10);

run;

proc phreg data=work_3;

model surv_time*group(0)=age NC LC alb chol CRP kangning

/risklimits;

ods output parameterestimates=output_mult;

run;

data work4;set work_3;

id=_n_;

ratio=ft3/ft4;

if age lt 65 then age_grp=1;else age_grp=2;

if nc lt 5.6 then nc_grp=1;else nc_grp=2;

if lc lt 1.7 then lc_grp=1;else lc_grp=2;

if chol lt 4.3 then chol_grp=1;else chol_grp=2;

if crp lt 6.7 then crp_grp=1;else crp_grp=2;

if alb lt 34.8 then alb_grp=1;else alb_grp=2;

if kangning=1 then kn_g=1;else kn_g=2;

/*if fpg lt 6.1 then diabetes=2;else diabetes=1;*/

/*if smoke=1 then smoke_g=1;else smoke_g=2;*/

/*if drink=1 then drink_g=1;else drink_g=2;*/

/*if xuezhi=1 then xuezhi_g=1;else xuezhi_g=2;*/

/*if sex=1 then gender=1;else gender=2;*/

/*if sbp gt 120 or dbp gt 80 then hyper=1;else hyper=2;*/

drop age chol crp;

rename age_grp=Age nc_grp=Neutrophils lc_grp=Lymphocytes chol_grp=Chol crp_grp=CRP;

run;

PROC LCA DATA=work4 OUTPARAM=out1 OUTSTDERR=out2 OUTSEEDS=out3 outpost=out4 outest=out5;

NCLASS 2;

ITEMS age Neutrophils Lymphocytes chol crp;

CATEGORIES 2 2 2 2 2;

ID ID;

SEED 100000;

/*COVARIATES ;*/

RHO PRIOR = 1;

/*BETA PRIOR = 1;*/

NSTARTS 50;

RUN;

%univariate1(train=work4,variable=ratio,time=surv_time,result=group,n=1);

proc sort data=out4(keep=id best);by id;run;

proc sort data=work4;by id;run;

data work5;merge out4 work4;by id;run;

proc freq data=work4;tables xinfangcd kangning;run;

proc means data=work4 median p25 p75 maxdex=1;var danhe hb wbc talb alb ft3 ft4 pt danhongsu zhijdanhs;run;

ods listing close;

ods rtf file='D:\Desk\hou.rtf' startpage=never;

%cli_frqtabs(data=work5,var=age Neutrophils Lymphocytes chol crp,class=best,col2=C1,col3=C2,format=8.1);

%cli_frqtabs(data=work5,var=xinfangcd kangning ,class=best,col2=C1,col3=C2,format=8.1);

%mgcomp(data=work5,var=danhe hb wbc talb alb ft3 ft4 pt danhongsu zhijdanhs,class=best,where=,prn=,dec=1,index=,del=1,tjl=0);

%cli_frqtabs(data=work5,var=sex,class=best,col2=C1,col3=C2,format=8.1);

%cli_frqtabs(data=work5,var=sex toast hbp DM xuezhi xinfangcd smoke drink

platet jaingzhiy kangning,class=best,col2=C1,col3=C2,format=8.1);

ods listing;

ods rtf close;

%univariate(train=work5,variable=best,ref=2,time=surv_time,result=group,n=1);

%univariate(train=work5,variable=best,ref=2,time=surv_time2,result=death,n=1);

%INCLUDE "D:\Desk\SAS \Ǳ ģ \SAS Graphics Macros for PROC LCA\LcaGraphicsV2\LCAGraphicsV2.sas";

%ItemResponsePlot(ParamDataset=out1);

/*Class1:Elderly With Low LC*/

/*Class2:Participants With Low NC and High LC*/

proc phreg data=work5;

class best(ref='2');

model surv_time*group(0)=best xinfangcd hb talb alb ft3 ft4

pt kangning zhijdanhs/risklimits;

ods output parameterestimates=output_mult;

run;

proc format;

value group

1="Class1"

2="Class2"

;

run;

ods html gpath="G:\file\desk" dpi=600;

ods graphics /width=14cm height=10cm

outputfmt=jpg;

PROC LIFETEST data=work5

plots=s( test atrisk( outside maxlen=13)=0 to 5 by 1 nocensor) maxtime=5;

time surv_time*group(0);

strata best / order=internal;

format best group.;

label best="Risk group" surv_time="Time(months)";

run;

%let yOptions = label="Survival"

linearopts=(viewmin=0.8 viewmax=1

tickvaluelist=(.8 .85 .90 .95 1.0));

%CompileSurvivalTemplates

PROC LIFETEST data=work5

plots=s( test atrisk( outside maxlen=13)=0 to 1 by 0.2 nocensor) maxtime=1;

time surv_time2*death(0);

strata best / order=internal;

format best group.;

label best="Risk group" surv_time2="Time(years)";

run;
